# FOXK1 interaction with FHL2 promotes proliferation, invasion and metastasis in colorectal cancer

**DOI:** 10.1038/oncsis.2016.68

**Published:** 2016-11-28

**Authors:** M Wu, J Wang, W Tang, X Zhan, Y Li, Y Peng, X Huang, Y Bai, J Zhao, A Li, C Chen, Y Chen, H Peng, Y Ren, G Li, S Liu, J Wang

**Affiliations:** 1 Guangdong Provincial Key Laboratory of Gastroenterology, Department of Gastroenterology, Nanfang Hospital, Southern Medical University, Guangzhou, China; 2 Center for Reproductive Medicine, Nanfang Hospital, Southern Medical University, Guangzhou, China; 3 Department of Rheumatism, Nanfang Hospital, Southern Medical University, Guangzhou, China; 4 Department of Pathogen Biology, School of Public Health and Tropical Medicine, Southern Medical University, Guangzhou, China; 5 Department of General Surgery, Nanfang Hospital, Southern Medical University, Guangzhou, China

## Abstract

The transcriptional factor Forkhead box k1 (FOXK1) is a member of the FOX family. The abnormal expression of FOXK1 may have an important role in tumour development. Our previous studies showed that four-and-a-half LIM protein 2 (FHL2) is a critical inducer of the epithelial-to-mesenchymal transition (EMT) and invasion. However, the molecular mechanism by which FOXK1 synergizes with FHL2 tumour proliferation, EMT and metastasis is not well defined. We evaluated that messenger RNA (mRNA) and protein expression levels by quantitative RT–PCR, western blot, immunofluorescence and immunohistochemistry (IHC) assays. The migration and invasive abilities of colorectal cancer (CRC) cells were evaluated using short hairpin RNA (shRNA)-mediated inhibition *in vitro* and *in vivo*. We showed that FOXK1 expression was upregulated in CRC compared with matched normal tissues. FOXK1 physically interacts with FHL2 in CRC. Moreover, higher expression levels of the two proteins were significantly associated with differentiation, lymph node metastasis, AJCC stage and poorer prognosis. Furthermore, the overexpression of FOXK1 in CRC cells is associated with EMT, invasion and metastasis. However, the siRNA-mediated repression of FHL2 in FOXK1-overexpressing cells reversed EMT and both the proliferative and metastatic phenotypes *in vitro* and *in vivo*. These data identified that the co-expression of FOXK1 and FHL2 enhances cell proliferation and metastasis through the induction of EMT. Thus, FOXK1 and FHL2 may serve as putative targets in the combined therapy of CRC.

## Introduction

The Drosophila transcription factor forkhead and subsequent mammalian orthologues of the forkhead DNA-binding domain were discovered over two decades ago.^1^ Forkhead transcription factors encode a subgroup of helix-turn-helix proteins.^2^ The arrangement of loops connecting the strands that flank one of the three helices gives rise to a butterfly-like appearance (hence, these proteins are termed ‘winged-helix' transcription factors).^3^ Through the transcriptional control of gene expression, many FOX protein members have important roles in the embryonic development,^4^ organogenesis and the regulation of a variety of physiological processes, such as cell cycle progression,^5^ cell survival^6^ and immune responses.^7^ Consequently, the dysregulation of the functions, subcellular localization and expression of FOX transcription factors leads to the development and progression of diseases, especially cancer.^8, 9^ For example, in the FOX family proteins, FOXM1 has been reported in several malignant tumours, including those of the breast,^10^ liver,^11^ pancreas,^12^ ovarian,^13^ lung^14^ and colon.^15^

Forkhead box k1 (FOXK1) is a member of the FOX transcription factor family and binds to a DNA consensus sequence (5′-WRTAAAAYA-3′) to regulate transcription.^16, 17^ The human *FOXK1* gene encodes predicted proteins most homologous to the mouse myocyte nuclear factor MNF/Forklead box K1 (Foxk1). The mouse version of FOXK1, Foxk1/MNF, exists as two isoforms, MNFa and MNFb, which differ through their alternative splicing leading to the production of the C-terminally truncated MNFb isoform.^18, 19^ The human FOXK1, the protein feature analysis predicted a forkhead domain, an FHA domain and a nuclear localization.^16^ Recently, we found that FOXK1 was overexpressed in 16 types of cancerous human tissues and appeared to have a crucial role in the development and progression of human carcinomas.^20^

Four-and-a-half LIM domains protein 2 (FHL2) is the second member of a small family of five proteins with four-and-a-half LIM domains.^21, 22^ This domain is a specialized double zinc finger (ZF) protein motif with versatile cellular roles as regulators of gene expression, cyto-architecture, cell adhesion, cell motility and signal transduction.^23, 24, 25^ Accumulated evidence indicate that FHL2 functions as an oncogene in some type of cancers.^22, 26, 27^ In a previous study, Shi *et al.*^28^ reported that FOXK1 interacts with FHL2 in the myogenic progenitor cell. However, the effects of the interaction of FOXK1 and FHL2 on the development, progression and prognosis of colorectal cancer (CRC) remain to be defined.

In the present study, we demonstrated that the expression of FOXK1 and FHL2 is significantly elevated in CRC tissues. Moreover, a high expression of both FOXK1 and FHL2 predicts poor prognosis in CRC patients. In addition, the co-expression of FOXK1 and FHL2 promotes the proliferation, invasion and metastasis both *in vitro* and *in vivo* in CRC cells.

## Results

### FOXK1 expression is higher in human CRC tissues

**Figure 1:**
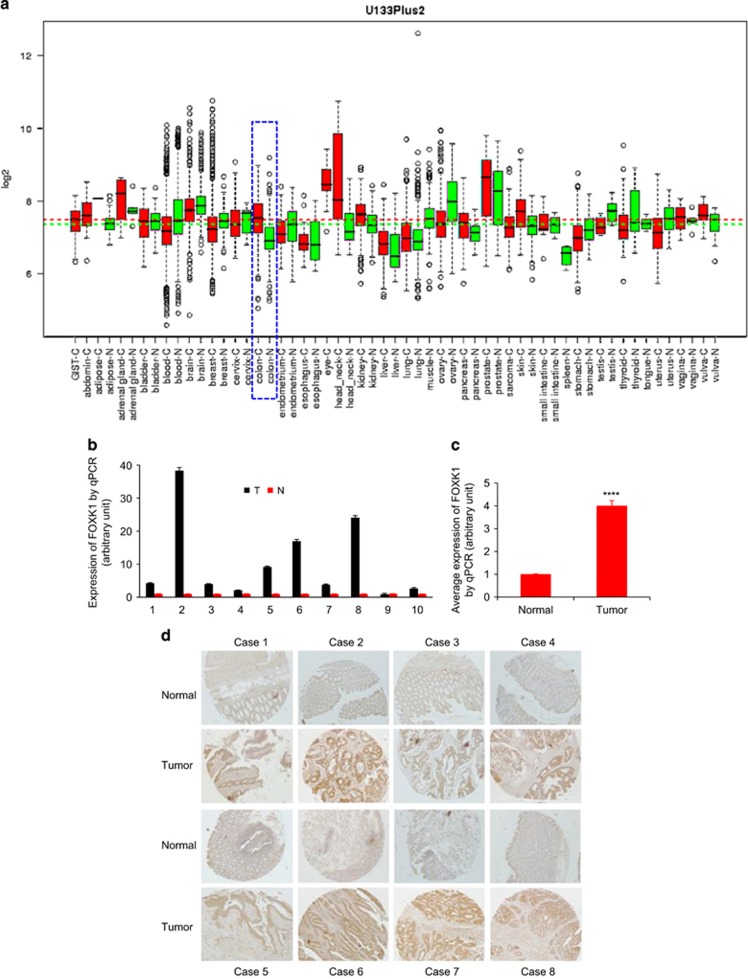
FOXK1 expression is higher in human CRC Tissues. (**a**) Expression pattern of *FOXK1* mRNA in normal and tumour tissues. *FOXK1* mRNA expression in various types of cancer was searched in the GENT database (available at http://medical-genomics.kribb.re.kr/GENT/). Boxes represent the median and the 25th and 75th percentiles; dots represent outliers. Red boxes represent tumour tissues; green boxes represent normal tissues. Red and green dashed lines represent the average value of all tumour and normal tissues, respectively. The asterisk indicates the significant increase of *FOXK1* expression in colon tumours compared with normal tissues. *FOXK1* mRNA expression of colon tissue: blue dotted lines. (**b**) Expression of FOXK1 by qRT–PCR in 10 pairs of colon cancer (tumour) and matched non-cancerous colonic tissues (normal). All of these experiments were repeated three times with identical findings. (**c**) On average, higher expression level of *FOXK1* was found in tumour than in normal tissues (*n*=10). ****P<0.001. (**d**) In eight selected cases, higher expression of *FOXK1* in tumour tissues was confirmed by immunohistochemistry (*n*=8). Scale bars, 200 μm in **d**.

In the GENT database, FOXK1 is upregulated in cancers of the adrenal gland, head neck, kidney, liver, lung, pancreas, skin, vulva and colon compared with corresponding normal tissues (Figure 1a). This finding suggests that FOXK1 may be associated with various types of cancer, including colon cancer.

We then examined the expression of FOXK1 in 10 pairs of human colon cancer tissues and matched non-cancerous colonic mucosa by qRT–PCR. As shown in Figure 1b, the majority (9/10 or 90%) of cancer tissues (T) exhibited a higher expression level of FOXK1 relative to their corresponding non-cancerous controls (N; Figure 1b). Figure 1c shows that the average expression of FOXK1 mRNA was ~4-fold higher in tumour tissues than in normal tissues. Higher expression levels of FOXK1 protein in colon cancer tissues were also confirmed by IHC (Figure 1d).

These data confirm that FOXK1 is overexpressed in CRC tissue.

### FOXK1 physically interacts with FHL2 in CRC

**Figure 2:**
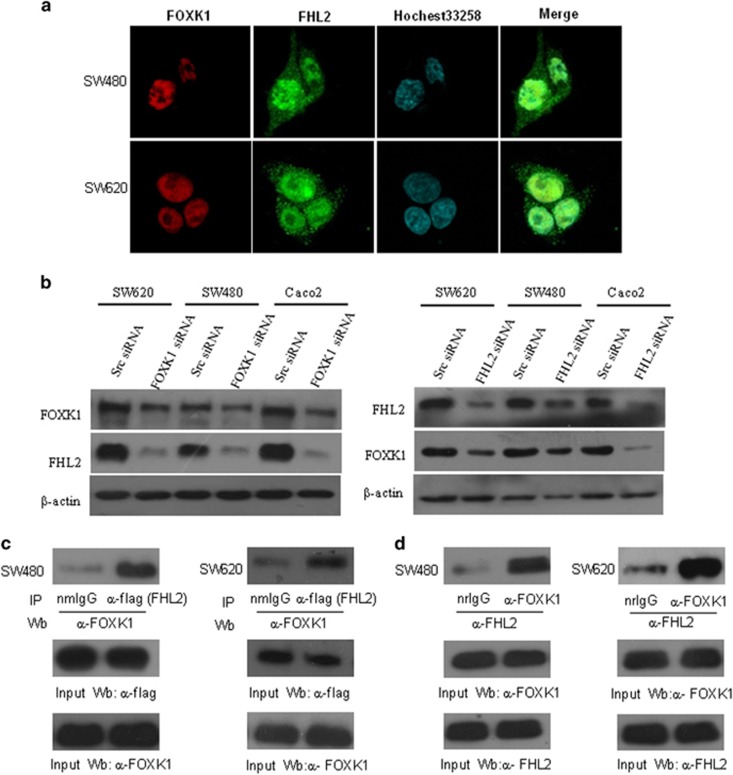
Interaction between FHL2 and FOXK1 proteins in CRC cells. (**a**) Double staining of FHL2 and FOXK1 in SW480 cells with Hoechst by confocal microscopy. (**b**) Western blotting analysis of FOXK1 and FHL2 expression in the indicated CRC cells. (**c**) PCI-flag-FHL2 plasmid was transfected into SW480 and SW620 cells. Immunoprecipitation was performed with anti-flag antibody, and pre-immune normal mouse immunoglobulin G (nm IgG) was used as control. Western blotting was performed with anti-FOXK1 antibody. The IP blot was probed with indicated antibodies to show the input of whole-cell lysates. IP, immunoprecipitation; Wb, western blot. (**d**) Cell lysates of SW480 and SW620 cells were immmunoprecipitated by anti-FOXK1 antibody or the control antibody, normal rabbit immunoglobulin G (nr IgG). Western blotting was carried out with anti-FHL2 antibody. The IP blot was probed with indicated antibodies to show the input. All these experiments were repeated two to three times with similar findings. Scale bars represent 20 μm in **a**.

Because FHL2 has been previously implicated in cancer cell growth and metastasis,^22, 24^ we investigated whether a correlation exists between FHL2 and FOXK1 expression in CRC. We first clarified the cellular distribution of the two proteins. A two-colour immunofluorescence assay showed that the endogenous FHL2 and FOXK1 proteins localized in the nuclei than the cytoplasm of SW480 and SW620 cells. A merged signal indicates the co-localization of the two proteins (Figure 2a). Second, we found that the downregulation of FOXK1 decreased FHL2 expression, whereas downregulation of FHL2 decreased FOXK1 expression in three colon cancer cell lines (Figure 2b).

It has been reported that FOXK1 is capable of interacting with FHL2 in myogenic progenitors,^28^ which suggests that a link between FOXK1 and FHL2 in CRC might exist. Third, we confirmed the interaction between FOXK1 and FHL2 proteins in the transient transfection of full-length flag-tagged FHL2 into SW480 and SW620 cells. Co-immunoprecipitation showed that FOXK1 could be co-precipitated with flag-tagged-FHL2 of the CRC cell line (Figure 2c). To further verify that the FHL2–FOXK1 interaction occurs with the endogenous FHL2, whole-cell lysates from SW480 and SW620 cells were prepared for immunoprecipitation. Indeed, the endogenous FHL2 was also capable of binding to FOXK1 (Figure 2d). Collectively, these findings suggest that FHL2 can physically interact with FOXK1.

### Co-expression of FOXK1 and FHL2 is associated with adverse prognosis in primary CRC

**Figure 3:**
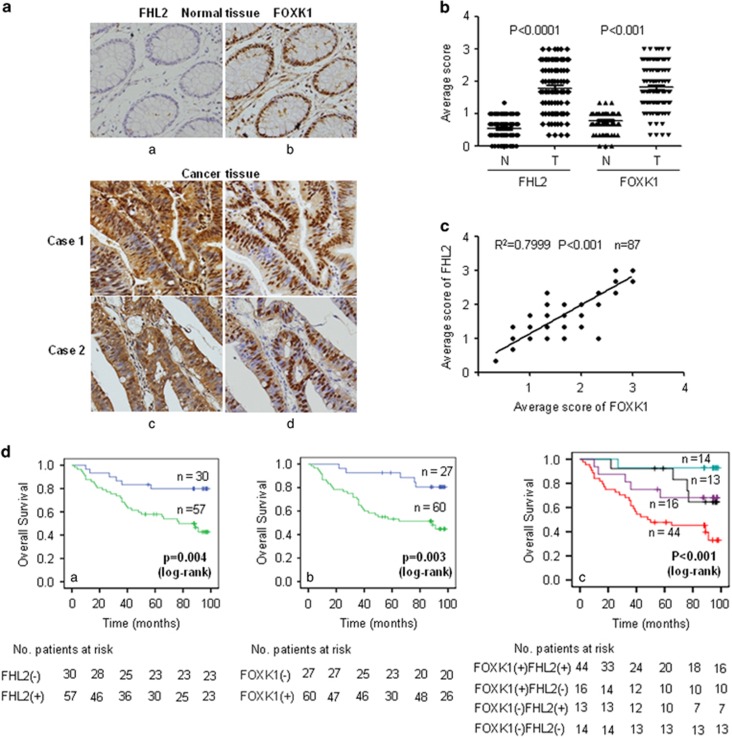
Positive correlation between FHL2 and FOXK1 expression in CRC. (**a**) FHL2 (a and c) and FOXK1 (b and d) expression in normal or cancerous colorectal tissue specimens were detected by IHC assays. These figures were the representatives of colorectal tissues from 87 cancerous and non-cancerous patients. Normal mouse IgG was used as the isotype control for the first antibody (a and b). (**b**) Average scores of the two proteins in normal and cancerous CRC tissues. ****P<0.001 between normal and cancer tissues. (**c**) FHL2 and FOXK1-positive staining were quantified and a Spearman correlation was performed. ****P<0.001. (**d**) Kaplan–Meier overall survival analysis of CRC patients. Survival analysis was performed according to the expression status of FHL2 (a), FOXK1 (b) and the combined expression status of FHL2 and FOXK1 (c), respectively. Scale bars represent 100 μm in **a**.

To investigate the relationship between FOXK1 and FHL2 in CRC, we examined their expression patterns in human CRC tissue. IHC was performed on serial sections of 87 CRC samples. We observed that FOXK1 and FHL2 had higher expression in cancer samples. FOXK1 located mainly in the nucleus, and FHL2 disseminated at both the nucleus and cytoplasm of the same cancer cells (Figure 3a). Semi-quantitative scoring of the two proteins showed that the expressions of both proteins in cancerous tissues were significantly higher than those found in adjacent normal colon tissues (Figure 3b). After calculating the regression coefficient between the expression scores of FOXK1 and FHL2, we observed a significant correlation between FOXK1 and FHL2 (Figure 3c) in CRC.

To explore the clinical relevance of FOXK1 and FHL2 expression, we analysed the clinicopathological features in CRC. Among these 87 patients, FOXK1 or FHL2 expression in tumour samples was found to be significantly correlated with tumour differentiation, lymph node metastasis, TNM stage, serosal invasion and tumour size; however, it was not correlated with gender, age or location (Supplementary Table 1).

To analyse the correlation between FHL2 (Figure 3d(a)) or FOXK1 (Figure 3d(b)) expression and the prognosis of CRC patients, Kaplan–Meier survival curves were generated. A high positive expression of each protein was correlated with poor outcome (Figure 3d(a) and (b)). Double-positive cases that expressed both proteins showed the worst prognosis (Figure 3d(c)). In multivariate analysis, tumour differentiation, AJCC stage, FOXK1 expression and FHL2 expression each had significant prognostic value for overall survival (Supplementary Table 2). Furthermore, when it was defined as a single factor, co-expression of FOXK1 and FHL2 was determined to be an independent prognostic factor (*P*=0.001; Supplementary Table 2).

### FOXK1 and FHL2 mutually promote proliferation and EMT phenotypes

**Figure 4:**
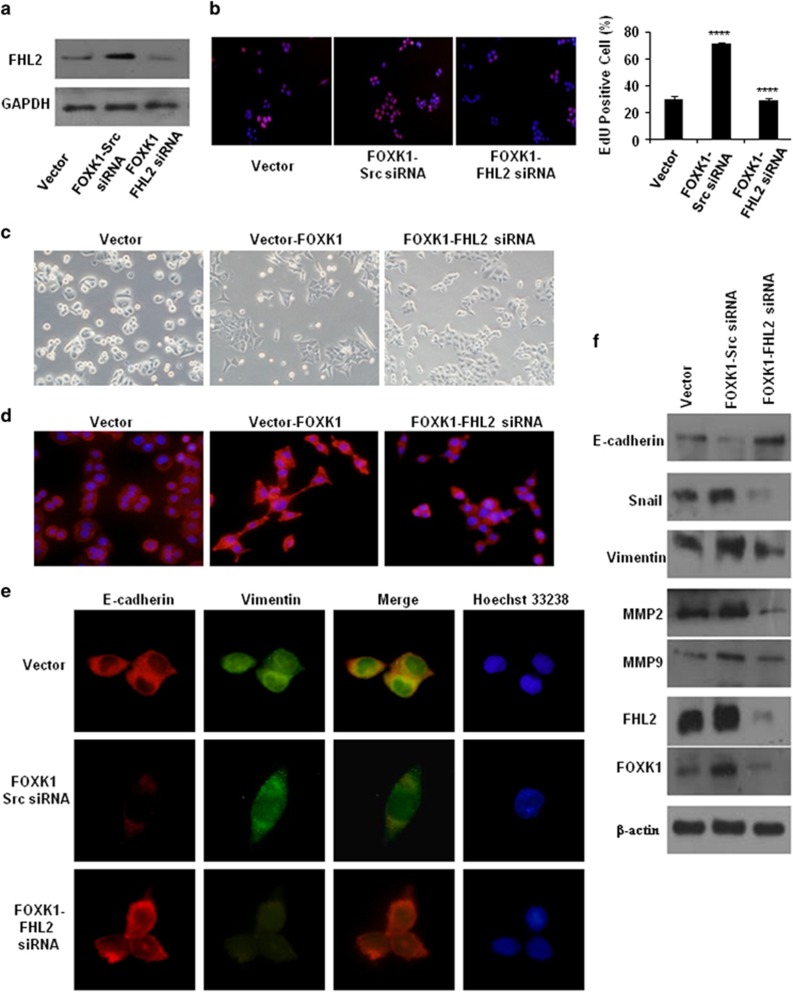
FOXK1 and FHL2 promote the proliferation and EMT in CRC cell. (**a**) Expression levels of FHL2 were detected by western blot analysis in SW480 cells, which were transfected with FOXK1 overexpressing plasmids, followed by transfection with FHL2 siRNA or Scr siRNA as a negative control. (**b**) SW480 stable transfectants of FOXK1, by transfection with FHL2 siRNA or Scr siRNA for 48 h, were subjected to the EdU incorporation assay; ****P<0.001. (**c**) The aberrant morphology of stably expressing FOXK1 transfected with FHL2 siRNA or src siRNA in SW480 cells, analysed by phase-contrast microscopy. (**d**) SW480 cells stained with rhodamine-phallotoxin for 48 h to identify F-actin filaments were visualized under fluorescent microscopy. (**e**) Immunofluorescence and microscopic visualization of E-cadherin (red) and vimentin (green) staining in Vector, FOXK1 src siRNA and FOXK1–FHL2-siRNA cells. (**f**) EMT biomarkers, including E-cadherin, vimentin, Snail, MMP2, MMP9, FOXK1 and FHL2, were detected by western blot 48 h after transfection. All the experiments were repeated three to four times with similar findings. Scale bars represent 100 μm in **b**, 20 μm in **c** and **d**, 10 μm in **e**.

We downregulated FHL2 in FOXK1-overexpressing cells using small interfering RNA (siRNA) and confirmed this effect by western blot (Figure 4a). FOXK1 promoted CRC cell proliferation, whereas FHL2 knockdown could inhibit growth in the FOXK1-overexpressed group in EdU incorporation assay (Figure 4b).

We then examined the morphologic features of these cells. The stable vector transfectants displayed a round or flat morphology with a short cytoplasmic process. However, the FOXK1 transfectants exhibited a spindle-like, fibroblastic morphology, which is one of the main characteristics of EMT. Long or dendritic-like cytoplasmic processes were visible under a phase-contrast microscope. FHL2 knockdown in FOXK1 overexpressing cells led to EMT reversion (Figure 4c). To further characterize FOXK1, we stained F-actin using phalloidin staining. Compared with the empty vector-expressing cells, the stable high expression of SW480 of the FOXK1 cell was present throughout the cytoplasm and at the rim zone of the protrusion (Figure 4d). FHL2 knockdown in FOXK1-overexpressing cells led to the mesenchymal to epithelial transition (MET) process, which is the reverse of the EMT process. Immunofluorescence staining of E-cadherin and vimentin confirmed the EMT-associated shift in marker expression (Figure 4e). Moreover, the expression of a typical EMT epithelial marker, E-cadherin, was upregulated after the knockdown of FHL2 in FOXK1-overexpressing cells. In contrast, the mesenchymal markers MMP2, MMP9, Vimentin and Snail were all downregulated (Figure 4f). Together, these data suggested that the FOXK1 and FHL2 promote the proliferation and EMT in CRC cells.

### FOXK1 cooperates with FHL2 and promotes migration and invasion of CRC cells

**Figure 5:**
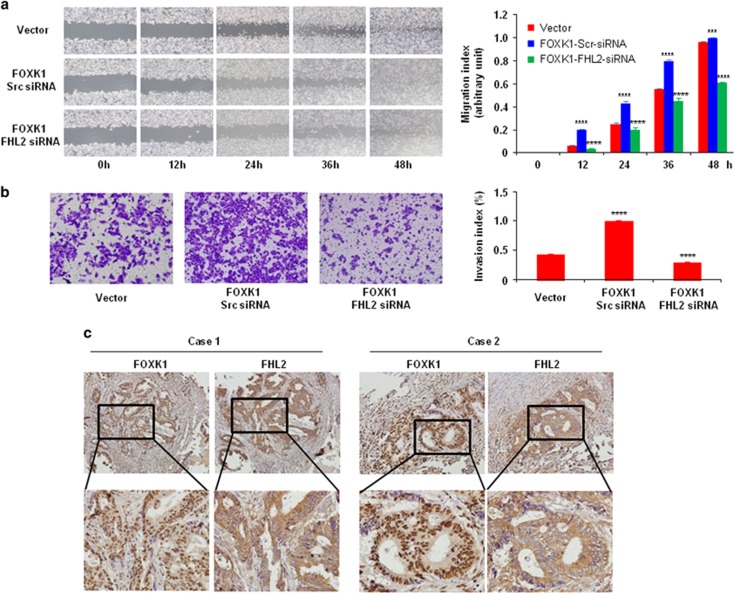
Co-expression of FOXK1 and FHL2 is associated with metastatic phenotypes in human CRC. (**a**) For the wound-healing experiments, the cells were analysed with live-cell microscopy. Original magnification, × 10. ***P<0.01, ****P<0.001. (**b**) Vector, stable FOXK1 transfectants were transfected with FHL2 siRNA 48 h later, and the invasive ability of the cells decreased; ****P<0.001. The experiments were repeated at least three times. (**c**) Representative IHC images are shown for FHL2 and FOXK1 expression in lymph node metastatic cancer tissues. Scale bars, 100 μm in **c**. These pictures were representatives of three independent experiments with identical results.

In the wound assay, the knockdown of FHL2 in FOXK1-overexpressing cells led to a decrease in the migratory potential of FOXK1-overexpressing cells *in vitro* (Figure 5a). Similarly, FHL2 downregulation in FOXK1-overexpressing cells decreased the invasion potential of FOXK1-overexpressing cells by 34.5% (Figure 5b).

Next, we detected the expression of FOXK1 and FHL2 in regional lymph nodes related with metastasis. In total, 26/33 of the metastatic tissues taken from lymph nodes highly expressed FOXK1 and FHL2 by means of IHC, as exemplified in two patients (Figure 4b). This result confirmed the positive correlation between FOXK1 and FHL2 by IHC. Together, these data suggest that the FOXK1–FHL2 axis promotes the invasion and metastasis of CRC cells.

### FOXK1 synergizes with FHL2 to promote tumour proliferation and metastasis *in vivo*

**Figure 6:**
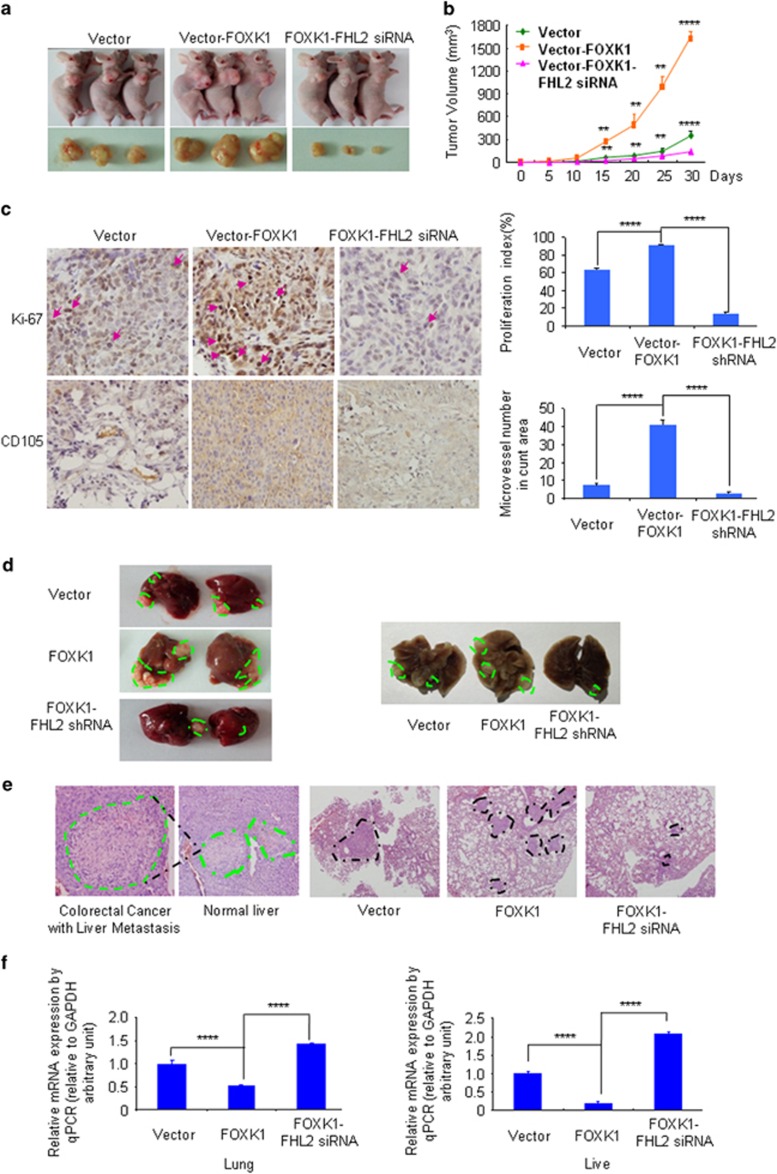
FOXK1 synergizes with FHL2 to promote tumour proliferation and metastasis *in vivo.* (**a**) Evaluation of tumorigenesis in nude mice subcutaneously injected with SW480-Vector, SW480-FOXK1 and SW480-FOXK1–FHL2-shRNA cells. Images were captured on day 30 after injection. (**b**) Tumour size was measured five days after tumour cell inoculation in each group. **P<0.05, ****P<0.001, vector vs FOXK1 and FOXK1 vs FOXK1–FHL2-shRNA, respectively. (**c**) FHL2 knockdown significantly inhibited FOXK1-induced proliferation (Ki-67, ****P<0.001, vector vs FOXK1 and FOXK1 vs FOXK1–FHL2-shRNA, respectively), and a considerable decrease of tumour vessel density (CD105, ****P<0.001, vector vs FOXK1 and FOXK1 vs FOXK1–FHL2-shRNA) was observed by IHC. (**d**) Mice were orthotopically transplanted with indicated cells (*n*=3 in each group). Representative images of metastatic loci in lungs or liver of blue dotted lines were shown. (**e**) The mice were killed, and metastatic cancer tissues were stained with haematoxylin and eosin (H&E). (**f**) The expression of E-cadherin in tumours derived from SW480 cells was determined by qPCR. ****P<0.001, vector vs FOXK1; FOXK1 vs FOXK2-FHL2-siRNA. Scale bars represent 100 μm in **c** and 200 μm in **d**.

To evaluate the impact of FOXK1 cooperating with FHL2 on tumour growth *in vivo*, we inoculated vector, FOXK1 stable transfectants and Lenti-FOXK1–FHL2-shRNA into BALB/c-*nu*/*nu* mice, as shown in Figure 6a. The tumour volumes of the FOXK1-overexpressed cells were markedly larger than those of the vector. FOXK1 overexpression progressed from a pronounced increase in vector cells at day 15 to a 4.4-fold increase in cancer area 30 days after injection (Figure 6b). On the contrary, tumours derived from the downregulation of FHL2 in FOXK1-overexpressed cells were markedly smaller than those of the FOXK1-treated mice from 15 to 30 days (Figure 6b). FHL2 knockdown inhibited the proliferation of FOXK1-overexpressed CRC SW480 cells *in vivo*.

We next examined the expression of cell proliferation markers (Ki-67) and angiogenesis markers (CD105) at the protein level in xenograft tumours. Representative images of tumour by IHC are shown in Figure 6c. The FOXK1 stable transfectants group showed a significant increase in the proliferation rate and tumour vessels compared with those observed in the vector group, whereas the knockdown of FHL2 inhibited the growth rate and tumour vessels in the FOXK1-overexpressed group.

To test the role of FOXK1 cooperating with FHL2 in progression, we injected into nude mice to examine the liver and lung metastases. FOXK1-overexpressing SW480 cells, but not control SW480 cells, formed a variety of large metastatic nodules in the liver and lung (Figure 6). Compared with the FOXK1-overexpressing cells, the FHL2 downregulation observed in FOXK1-overexpressing cells led to a significant reduction of visible tumours in the liver and lung, which correlated to a lower number of metastasis loci (Figure 6d). The presence of metastasis from CRC to the liver and lung was confirmed by histological analysis (Figure 6e).

To further demonstrate whether FOXK1 cooperating with FHL2 correlates with EMT, the expression of FHL2 was repressed in FOXK1-overexpressing cells at the mRNA level in orthotopic xenograft tumours. The overexpression of FOXK1 resulted in a significant loss of epithelial marker E-cadherin, whereas the downregulation of FHL2 in FOXK1-overexpressed cells caused an increase in E-cadherin (Figure 6f).

Taken together, these results clearly indicated that the FOXK1–FHL2 axis has an important role in development and metastasis during CRC.

## Discussion

In this study, we demonstrated that FOXK1 physically interacts with FHL2 and thus contributes to the EMT, invasion and migration of CRC. The present data indicated that patients with high FOXK1 or FHL2 expression had a poorer prognosis than did those with low FOXK1 or FHL2 expression. In particular, patients with a high co-expression of FOXK1 and FHL2 had a shorter median survival than did those without. Therefore, these findings suggest that the cooperative relationship between FOXK1 and FHL2 has a pivotal role in CRC. FOXK1 is a transcription factor that belongs to the forkhead family, which consists of the winged-helix DNA-binding domain and the N-terminal and C-terminal transcriptional domains.^16, 17, 20^ Wang *et al.*^29^ reported that the FOXK1 protein levels are elevated in human CRC and positively regulate Wnt/b-catenin by translocating DVL into the nucleus. FHL2 is a member of the four-and-a-half-LIM protein (FHL) family. The LIM domains are double zinc finger motifs that have multiple roles in protein–protein interactions, such as functional modifiers and adaptors.^21, 22, 26^ Shi *et al.*^28^ discovered that Fhl2 interacts with Foxk1 to form a complex that represses the Foxo4 transcriptional activation of p21 in the myogenic progenitor cell population. However, the clinical implications of FOXK1 and FHL2 co-expression and prognosis of patients with CRC have not been investigated. Here, we revealed that the distribution pattern of FOXK1 was highly congruent with that of FHL2 and that FOXK1 protein expression was highly correlated with FHL2 expression. The co-expression of FOXK1 and FHL2 was significantly correlated with differentiation and lymph node metastasis. Further survival analysis indicated that the overexpression of FOXK1 and/or FHL2 predicted a poor prognosis. Thus, our study further confirmed that FOXK1 and/or FHL2 could be used as an unfavourable prognostic biomarker for CRC patients.

The function of FHL2 in oncogenesis is still controversial. In rhabdomyosarcoma cells, the overexpression of FHL2 induced apoptosis.^30^ In tongue squamous cancer cells, however, the expression of FHL2 contributed to growth, proliferation, invasiveness and metastasis.^31^ In prostate cancer cells, the expression of FHL2 alone also inhibited FOXO1-induced apoptosis.^32^ In gastric and colon cancer cells, the suppression of FHL2 inhibited the serum-dependent, anchorage-dependent and anchorage-independent cell growth.^22^ Most of the previous studies on rgw overexpression of FHL2 found that it enhanced cell proliferation and migration. Thus, it appears that FHL2 can regulate tumorigenesis in multiple human cancers. The knockdown of FOXK1 resulted in decreased cell proliferation rates and the development of the malignant phenotype in human osteosarcoma U2OS cells.^33^ The data indicate that the synergistic overexpression of FHL2 and FOXK1 enhanced cell growth. Consistently, we revealed that FHL2 knockdown resulted in a marked blockage of FOXK1 expression *in vitro* and *in vivo*. Therefore, we speculated that the co-expression of FOXK1 and FHL2 might have a role in the development of CRC.

The epithelial–mesenchymal transition (EMT) is a cellular mechanism that has been recognized as a central feature of normal tissue development.^34, 35, 36^ During cancer progression, advanced tumour cells frequently exhibit a conspicuous downregulation of epithelial markers and a loss of intercellular junctions, resulting in a loss of epithelial polarity and reduced intercellular adhesion. These alterations are often accompanied by increased cell motility and the expression of mesenchymal-specific proteins.^24, 27^ Therefore, EMT can promote hallmark features of carcinoma that correlate with poor histologic differentiation, destruction of tissue integrity, invasion and metastasis. In a previous study, we found that FHL2 downregulation in FOXK1-overexpressing cells was positively associated with the low expression of vimentin but negatively associated with high E-cadherin expression. In addition, the overexpression of two proteins, that is, MMP2 and MMP9, was correlated strongly with the expression of Snail, a central transcription factor as E-cadherin repressor. Similar results were obtained in the current study, as the FOXK1 cells often exhibited a fibroblast-like, spindle-shaped phenotype, whereas FHL2 knockdown in FOXK1-overexpressing cells led to EMT reversion. Taken together, these results strongly suggest that FOXK1 and FHL2 are mutually essential for maintaining EMT and metastatic phenotypes.

In summary, we have identified that the co-expression of FOXK1/FHL2 in CRC could be a critical factor in predicting disease progression and clinical outcomes. In addition, FOXK1 and FHL2 are mutually essential for maintaining EMT and metastatic phenotypes. Therefore, FOXK1 and FHL2 may be putative targets in the combined therapy of CRC.

## Materials and methods

### Reagents, cells and culture conditions

Rhodamine-phallotoxin was purchased from Molecular Probes (Eugene, OR, USA). Mouse anti-human FHL2 used for western blot were a product of the MBL international incorporation (11–134, MBL International Incorporation, Woburn, Japan). Rabbit anti-human FHL2 antibody was used for IHC and was purchased from Abcam (Cambridge, UK). Mouse anti-FOXK1 (G-4), Vimentin (E-5), E-Cadherin (H-108), GAPDH (G-9) and bovine anti-mouse IgG-TR and goat anti-rabbit IgG-FITC were purchased from Santa Cruz (Santa Cruz, CA, USA). Mouse anti-human Flag were a product of Sigma (St Louis, MO, USA). Goat anti-rabbit immunoglobulins/HRP, rabbit anti-mouse immunoglobulins/HRP, normal mouse and rabbit IgG were all products of Dako (Carpinteria, CA, USA).

Human colon cancer cell lines SW480, Caco2 and SW620 were all maintained in our laboratory as previously described.^26, 27^ The cells were cultured in RPMI 1640 (Life Technologies, Inc., Gaithersburg, MD, USA) supplemented with 10% fetal bovine serum, 100 μg/ml streptomycin and 100 units/ml penicillin in a humidified incubator at 37 °C with an atmosphere of 5% CO_2_.

### Constructs, establishment of stable transfectants and siRNA transfection.

The FOXK1 open reading frame and 3′-untranslated region were cloned into pcDNA 3.1(+) in a previous study.^20^ FLAG-tagged constructs FHL2 (76) in PCI 3XFLAG plasmids expressed the full length of the FHL2 protein.^24^ To establish stable cell lines, the cells were transfected with empty SW480-pcDNA 3.1 (vector), and SW480-pcDNA 3.1-FOXK1 were passaged at 1:15 (vol/vol) and cultured in RPMI 1640 medium supplemented with Geneticin (G418, Calbiochem, Darmstadt, Germany) at 800 μg/ml for 4 weeks. The siRNA sequences were as follows: FOXK1 sense strand, 5′-CGAAUCUCUCUUUGGCAAGdTdT-3′ the scrambled (src) siRNA 5′-TTCTCCGAACGTGTCACGT-3′, which does not target any gene, was used as the negative control. Forty-eight hours after transfection, the western blot analyses were performed 3 days after transfection.

#### Tissue multi-array

Tissue microarray human CRC tissues and matched non-cancerous colonic tissues were purchased from superchip (Shanghai, China). The Human ethics was provided and consent was obtained from all the subjects to publish their photos. In the current study, all 87 tissues were adenocarcinomas. The expression of FOXK1 and FHL2 was detected by IHC in tissue microarray slides. Tumour staging was defined according to the criteria for histological classification proposed by the International Union against Cancer. Tissue in which more than 10% of the cancer cells were positively stained was considered positive. Scoring of tissue slides was performed independently by two investigators. The percentage of positive cells and the intensity of staining were scored from 0 to 3: 0, <10% of cells stained; 1, 10–50% of cells stained; 2, 50–75% of cells stained; and 3, >75% of cells stained. The study was approved by the institutional human ethics committee of the relevant institutions.

### Western blot

The whole-cell lysates were prepared with lysis buffer (20 mm Tris-HCl, 1 mm EDTA, 1 mm EGTA, 1 mm sodium vanadate, 0.2 mm phenylmethylsulfonyl fluoride, 0.5% NP-40, 1 μg/ml leupeptin, 1 μg/ml aprotinin and 1 μg/ml pepstatin A). Protein concentrations were determined with BCA Protein Assay Kit (Pierce, Rockford, IL, USA). Equal aliquots of total cell lysates (30 μg) were solubilized in sample buffer and electrophoresed on denaturing SDS–PAGE gel (5% stacking gel and 8~12% separating gel). The proteins were then transferred to polyvinylidene difluoride membranes (Millipore, Bedford, MA, USA). The blots were probed with primary antibody followed by the HRP-conjugated secondary antibody. Antigen–antibody complexes were visualized by the enhanced chemiluminescence system (Amersham Biosciences, Little Chalfont Buckinghamshire, UK).

### EdU incorporation assay

Each group of isolated tumour cells was seeded onto 96-well plates in triplicate at a density of 2 × 10^3^ per well for 48 h of incubation. The cells were incubated for an additional 2 h in medium containing 50 μm EdU (RiboBio, Guangzhou, China). The cells were then washed with PBS, fixed and permeabilized with PBS containing 4% paraformaldehyde and 0.5% Triton X-100. The cells were incubated with 1 × Apollo reaction cocktail (100 μl/well) for 30 min. DNA was incubated with Hoechst 33342 stain (100 μl/well) for 30 min and visualized with an inverted fluorescence microscope (Leica DM5500, Wetzlar, Germany). For each EdU experiment, five random fields were imaged by × 100 magnification. Captured images were processed and analysed using the ImageJ software. The number of EdU-positive cells was identified by Hoechst nuclei staining and expressed as a percentage of the total number of cells in each field.

### Cell migration and invasion assays

Cell migration was assessed using a wound-healing assay. The cells that had been plated in six-well plates with 100% confluence were wounded with a pipette tip at time 0. The media were changed to remove cell debris, and the cells were cultured in the presence of 10 μg/ml mitomycin C to inhibit cell proliferation. Photographs were taken 48 h later.

Cell invasion was assessed using Matrigel invasion chamber (BD Biosciences, Franklin Lakes, NJ, USA), as per the protocol provided by the manufacture. Briefly, the siRNA transfections were resuspended in serum-free media. Then, 5 × 10^4^ cells were placed in each Transwell membrane filter inserts, the lower chamber was filled with 600 μl of complete medium, and the samples were incubated for an additional 24 h. Invasive cells were stained with 0.2% of crystal violet and counted under a microscope. The average number of cells in five fields per membrane was counted in triplicate inserts. The invasion index was expressed as the percentage of test cells to that of control cells or treatments.

#### RNA isolation and quantitative real-time RT–PCR

The cells were collected, and total RNA was extracted using TRIzol Reagent (Gibco BRL and Life Technologies, Grand Island, NY, USA). RNA was reversely transcribed to complementary DNA (cDNA) by Thermoscript RT system reagent (Gibco BRL) in accordance with the manufacturer's instructions.

Quantitative real-time PCR was performed using Applied Biosystems Sequence Detection System 7900 (ABI Prism 7900HT, Applied Biosystems Company, Foster City, CA, USA) with 10 μl mixture composed of Power SYBR GREEN PCR Master Mix (Applied Biosystems), 500 nmol of each primer, and 300 ng of cDNA templates. The reactions were carried out with initial denaturation at 95 °C for 5 min, followed by 60 cycles of 20 s at 94 °C, 20 s at 60 °C and 40 s at 72 °C. A final extension at 72 °C for 5 min was included before a temperature ramp from 72 °C to 95 °C at 0.1 °C/s with continuous fluorescent acquisition. Each cDNA sample was duplicated for each instance of quantitative RT–PCR and the average relative fold mRNA expression levels were determined using the 2^−^^ΔΔCt^ method with GAPDH being detected as the internal control. The primer sequences used in RT–PCR were as follows: FOXK1 forward: 5′-ACACGTCTGGAGGAGACAGC-3′ and reverse: 5′-GAGAGGTTGTGCCGGATAGA-3′ (196 bp); E-cadherin forward: 5′-TGGCACAAATCTGCAGTCTC-3′ and reverse: 5′-GTGTATGTGGCAATGCGTTC-3′ (200 bp); GAPDH forward: 5′-GTCAACGGATTTGGTCGTATTG-3′, and reverse: 5′-CTCCTGGAAGATGGTGATGGG-3′ (204 bp).

### Confocal microscopy

The cells grown in cover glass were fixed with 4% paraformaldehyde, and the nonspecific bindings were blocked by incubation with 1% BSA. The glasses were probed with the first antibodies followed by TR- (Texas red) or FITC-conjugated second antibodies. The nuclei were counterstained with 1 μg/ml Hoechst 22358 and sealed with nail varnish. The confocal images were captured with a Zeiss LSW 710 confocal microscope (Oberkochen, Germany) using the × 40 objectives.

### Co-immunoprecipitation

To precipitate the target proteins, the lysates of cells without or with stably transfection of tagged constructs were incubated with 3 μg of the first antibody for 3 h at 4 °C, followed by incubation with the precleared protein A-agarose bead (Roche, Mannheim, Germany) slurry. After extensive washing, the samples were subjected to western blot to detect the potential interacting proteins.

### Construction and transfection of lentiviral vectors

To further investigate the effects of the siRNA-induced downregulation of FHL2 in FOXK1-overexpressed cells on tumour growth *in vivo*, an FHL2-RNAi lentiviral vector (pGCSIL-FHL2 shRNA) was constructed (Shanghai GeneChem Co, Ltd, Shanghai, China). Double-stranded oligonucleotides encoding human FHL2-vshRNA (5′-CCGGCCGAATCTCTCTTTGGCAAGTCAAGAGCTTGCCAAAGAGAGATTCGTTTTTG-3′) were inserted into the shRNA expression vector pGCSIL (Shanghai GeneChem Co., Ltd.), and the identities of the clones were verified by sequencing.

A recombinant lentiviral vector was produced by co-transfecting HEK293T cells with the lentiviral expression vector and the packaging plasmid mix using Lipofectamine 2000. The viruses were harvested 48 h after transfection, and viral titre were determined. SW480 cells (1  ×  10^5^) were infected with 1 × 10^6^ recombinant lentivirus-transducing units in the presence of 6 μg/ml polybrene (Sigma).

### *In vivo* tumour growth assay

Tumour growth was evaluated in a nude mouse xenograft model. SW480 cells (5 × 10^6^) in 0.1 ml of RPMI, were inoculated subcutaneously into the right flanks of 5- to 6-week-old female BALB/c-nu/nu mice (three mice in each group, nine mice were allocated to three experiment groups randomly before the experiment, the investigator was blinded to the group allocation during the experiments and the method of randomization was used to process the mice; Laboratory Animal Unit, Southern Medical University, China; the decision of laboratory animal ethics number was L2015065), and the resulting tumour sizes were measured weekly. Institutional guidelines were followed for handling the animals. The mice were maintained under sterile conditions. The tumour volumes were calculated as follows: total tumour volume (mm^3^)=*L* × *W*^2^/2, where *L* is the length and *W* is the width. On day 30 after inoculation, the mice were killed, and the tumours were dissected and weighed. IHC analysis was performed using anti-Ki-67 and anti-CD105 antibodies.

### *In vivo* metastasis assays

The mice were anaesthetised with isoflurane (mice were allocated to experiment groups randomly). For the orthotopic tumour implantation assays, pcDNA 3.1, pcDNA 3.1-FOXK1 or lenti-FOXK1–FHL2-siRNA-expressing cells (1 × 10^6^ in 0.1 ml of PBS) were inoculated into the dorsal subcostal incision to expose the spleen. The volume (50 μl) of tumour cell suspension was slowly injected into the spleen using a 25-gauge needle. Thirty days later, all the mice were killed, individual organs were removed and metastatic tissues were analysed with haematoxylin and eosin staining.

### Statistical analysis

Quantitative data from the experiments with biological replicates were presented as the means (±s.d.). Student's *t*-tests analysis were used to analyse the differences between groups. Pearson's correlation efficiency analysis was also used. Survival analysis were performed via Kaplan–Meier and log-rank test. The differences were considered statistically significant at *P*<0.05.
