# Supplementary material for: FOXK1 interaction with FHL2 promotes proliferation, invasion and metastasis in colorectal cancer
Source: Oncogenesis. 2016 Nov 28;5(11):e271–. doi: 10.1038/oncsis.2016.68 (PMC5141290; doi:10.1038/oncsis.2016.68)
Supplement: Supplementary Tables [file oncsis201668x1.doc]

**Supplementary Table 1**. Correlation between FOXK1/FHL2 protein expression and the clinicopathological parameters of CRC

|  |  |  | | **Expression of FOXK1** | | |  | | **Expression of FHL2** | | | |  | |
| --- | --- | --- | --- | --- | --- | --- | --- | --- | --- | --- | --- | --- | --- | --- |
| **Features** | **Total number (n=87)** | | **Low** | | **High** | | | **P-value** | **Low** | **High** | | **P-value** | |  |
| **Age(years)** |  |  | |  | |  | 0.615 | |  | |  | | 0.397 | |
| **<60** | 19 | | 5(26.3%) | | 14(73.3%) | | |  | 59(26.3%) | 14(73.7%) | |  | |  |
| **>=60** | 68 | | 22(32.4%) | | 46(67.6%) | | |  | 25(36.8%) | 43(63.2%) | |  | |  |
| **Gender** |  |  | |  | |  | 0.761 | |  | |  | | 0.709 | |
| **Male** | 44 | | 13(29.5%) | | 31(70.5%) | | |  | 16(36.4%) | 28(63.6%) | |  | |  |
| **Female** | 43 | | 14(32.6%) | | 29(67.4%) | | |  | 14(32.6%) | 29(67.4%) | |  | |  |
| **Location** |  |  | |  | |  | 0.327 | |  | |  | | 0.482 | |
| **C,A,T** | 39 | | 10(25.6%) | | 29(74.4%) | | |  | 15(38.5%) | 24() | |  | |  |
| **D,S.R** | 48 | | 17(35.4%) | | 31(64.6%) | | |  | 15(31.2%) | 33(68.8%) | |  | |  |
| **Tumour size(cm)** | |  | |  | |  | 0.015 | |  | |  | | 0.027 | |
| **<3** | 5 | | 4(80%) | | 1(20%) | | |  | 4(80%) | 1(20%) | |  | |  |
| **>=3** | 82 | | 23(28%) | | 59(72%) | | |  | 26(31.7%) | 56(68.3%) | |  | |  |
| **Differentiation** | |  | |  | |  | 0.044 | |  | |  | | 0.017 | |
| **Well** | 11 | | 7(63.6%) | | 4(36.4%) | | |  | 8(72.7%) | 3(28%) | |  | |  |
| **Moderate** | 60 | | 16(26.7%) | | 44(73.3%) | | |  | 17(28.3%) | 43(71.7%) | |  | |  |
| **Poor** | 16 | | 4(25%) | | 12(75%) | | |  | 5(29.2%) | 11(70.8%) | |  | |  |
| **AJCC stage** | |  | |  | |  | 0.974 | |  | |  | | 0.652 | |
| **Ⅰ,Ⅱ** | 55 | | 17(30.9%) | | 38(69.1%) | | |  | 18(32.7%) | 37(67.3%) | |  | |  |
| **Ⅲ,Ⅳ** | 32 | | 10(31.2%) | | 22(68.8%) | | |  | 12(37.5%) | 20(62.5%) | |  | |  |
| **Lymph node metastasis** | | |  | |  | | | 0.012 |  |  | | 0.042 | |  |
| **N0** | 54 | | 22(40.7%) | | 32(59.3%) | | |  | 23(42.6%) | 31(57.4%) | |  | |  |
| **N1,N2** | 33 | | 5(15.2%) | | 28(84.8%) | | |  | 7(21.2%) | 26(78.8%) | |  | |  |
| **TNM stage** |  |  | |  | |  | 0.016 | |  | |  | | 0.003 | |
| **Ⅰ,Ⅱ** | 7 | | 5(71.4%) | | 2(28.6%) | | |  | 6(85.7%) | 1(14.3%) | |  | |  |
| **Ⅲ,Ⅳ** | 80 | | 22(27.5%) | | 58(72.5%) | | |  | 24(30%) | 56(70%) | |  | |  |
| **Serosel invasion** | |  | |  | |  | 0.004 | |  | |  | | 0.009 | |
| **No** | 6 | | 5(83.3%) | | 1(16.7%) | | |  | 5(83.3%) | 1(16.7%) | |  | |  |
| **Yes** | 81 | | 22(27.2%) | | 59(72.8%) | | |  | 25(30.9%) | 56(69.1%) | |  | |  |
| **Expression of FHL2** | |  | |  | |  | 0.022 | |  | |  | |  | |
| **Low expression 30** | |  | | 14(46.7%) | | 16(53.3%) |  | |  | |  | |  | |
| **High expression 57** | |  | | 13(22.8%) | | 44(77.2%) |  | |  | |  | |  | |

**Supplementary** Table 2. Univariate and multivariate analyses of different prognostic factors in 87 patients with CRC

|  |  | |  |  |
| --- | --- | --- | --- | --- |
|  | **Univariate analysis** | | **Multivariate analysis** | |
| **Features** | **HR (95%CI)** | **P-value** | **HR(95%CI)** | **P-value** |
| **Age (years)** | 1.427(0.594-3.428) | 0.427 |  |  |
| **<60** |  |  |  |  |
| **>=60** |  |  |  |  |
| **Gender** | 1.414(0.732-2.731) | 0.302 |  |  |
| **Male** |  |  |  |  |
| **Female** |  |  |  |  |
| **Location** | 1.306(0.668-2.852) | 0.435 |  |  |
| **C,A,T** |  |  |  |  |
| **D,S.R** |  |  |  |  |
| **Tumour size (cm)** | 23.046(0.107-4972.321) | 0.235 |  |  |
| **<3** |  |  |  |  |
| **>=3** |  |  |  |  |
| **Differentiation** | 2.813(1.511-5.235) | 0.001 | 2.307(1.129-4.716) | 0.022 |
| **Well** |  |  |  |  |
| **Moderate** |  |  |  |  |
| **Poor** |  |  |  |  |
| **AJCC Stage** | 2.267(1.178-4.361) | 0.014 | 2.183(1.096-4.348) | 0.026 |
| **A,B** |  |  |  |  |
| **C,D** |  |  |  |  |
| **Lymph node metastasis** | 2.497(1.296-4.812) | 0.006 |  |  |
| **No** |  |  |  |  |
| **Yes** |  |  |  |  |
| **TNM stage** | 1.232(1.041-2.507) | 0.037 |  |  |
| **Tis,T1,T2** |  |  |  |  |
| **T3,T4** |  |  |  |  |
| **Serosel invasion** | 2.8964(1.277-3.366) | 0.039 |  |  |
| **No** |  |  |  |  |
| **Yes** |  |  |  |  |
| **Expression of FOXK1** | 3.818(1.48-9.818) | 0.006 | 2.92（1.202-7.118） | 0.02 |
| **Low expression** |  |  |  |  |
| **High expression** |  |  |  |  |
| **Expression of FHL2** | 3.356(1.392-8.089) | 0.007 | 3.108（1.198-8.060) | 0.018 |
| **Low expression** |  |  |  |  |
| **High expression** |  |  |  |  |
| **Co-expression** | 2.019(1.343-3.036) | 0.001 | 2.021(1.32-3.095) | 0.001 |
| **FOXK1(high) FHL2(high)** |  |  |  |  |
| **FOXK1(high) FHL2(low)** |  |  |  |  |
| **FOXK1(low) FHL2(high)** |  |  |  |  |
| **FOXK1(low) FHL2(low)** |  |  |  |  |
